# *ITGA4* genetic variants as a predictor of therapeutic responsivity to autoimmune diseases

**DOI:** 10.3389/fphar.2025.1745315

**Published:** 2026-01-08

**Authors:** Matheus D. De Matos, Bárbara D. Guimenes, Giovanni K. Pavani, Gabriel Gripp Fernandes, Guilherme C. Montes, Fabrícia L. Fontes-Dantas

**Affiliations:** 1 NeuroPharmacoGenetics Laboratory, Department of Pharmacology and Psychobiology, Rio de Janeiro State University, Rio de Janeiro, Brazil; 2 Postgraduate Program in Biosciences, Rio de Janeiro State University, Rio de Janeiro, Brazil; 3 Postgraduate Program in Clinical and Experimental Physiopathology (FISCLINEX), Rio de Janeiro State University, Rio de Janeiro, Brazil; 4 Department of Biophysics and Physiology, Federal University of Juiz de Fora, Juiz de Fora, Minas Gerais, Brazil

**Keywords:** autoimmune diseases, integrin α4, ITGA4, multiple sclerosis, pharmacogenetics, therapeutic responsivity

## Abstract

Integrins are pivotal adhesion receptors that mediate dynamic communication between cells and their surrounding environment. Through bidirectional signaling, they regulate key physiological processes such as cell migration, inflammation, and tissue organization. Among them, the α4 integrin subunit, encoded by the *ITGA4* gene, forms α4β1 (VLA-4) and α4β7 heterodimers, both essential for leukocyte adhesion and migration across inflamed tissues and blood–brain barrier. Variability in therapeutic response to α4 integrin inhibitors, such natalizumab, highlights the need to clarify the genetic and molecular determinants that regulate *ITGA4* function. This mini review provides an updated overview of *ITGA4* from historical, biological, and pharmacogenetic perspectives. We summarize current evidence linking *ITGA4* genetic variants with disease susceptibility, progression, and therapeutic outcomes in autoimmune and neuroinflammatory disorders. Finally, we discuss existing limitations, population inconsistencies, and emerging strategies integrating multi-omic and *in silico* analyses to translate *ITGA4* variants into predictive biomarkers for personalized immunotherapy.

## 1 Introduction

Integrins are a major component of cell adhesion receptors that seem to be present in all Metazoans (Sebé-Pedrós et al., 2010). Beyond serving as adhesion molecules, integrins are central mediators of bidirectional communication between cells and their surrounding environment (Kadry and Calderwood, 2020). They regulate cell–cell and cell–matrix interactions, enabling cells to sense biochemical and mechanical cues from the extracellular matrix (ECM) and to respond appropriately (Berman and Kozlova, 2000). This dual role places integrins at the core of essential processes such as tissue organization, immune surveillance, wound healing, and inflammatory responses (Arana et al., 2008; Hogg et al., 2011; Mezu-Ndubuisi and Maheshwari, 2021; Pang et al., 2023).

The mechanotransduction is the ability of cells to convert mechanical signals from their environment into biochemical responses and is a pivotal function of integrins. In the context of integrin signaling, mechanical forces can modulate integrin conformation, clustering, and downstream signaling cascades, thus influencing key cellular behaviors (Cooper and Giancotti, 2019). Through their capacity to link the ECM to the actin cytoskeleton, integrins transmit mechanical forces that modulate gene expression, cytoskeletal dynamics, and cellular fate. Aberrant mechanotransduction has been implicated in pathological conditions, including fibrosis, cancer progression, and autoimmune diseases, where inappropriate mechanical signaling contributes to immune cell activation, infiltration, and tissue damage (Roca-Cusachs et al., 2012; Sun et al., 2016).

The integrin receptor signaling pathway involves either “inside-out” signaling, which alters the integrin’s conformational state and modulates its affinity for ECM ligands, or “outside-in” signaling, which activates the integrin through various signaling cascades triggered by multiple external stimuli such as ECM engagement, mechanotransduction, or signaling from non-ECM ligands resulting in cell survival, proliferation, and spreading (Pang et al., 2023). Inside-out signaling is initiated when a chemokine binds to a G protein-coupled receptor (GPCR) on the cell membrane, triggering a cascade that converts Rap1-GDP into Rap1-GTP. Rap1-GTP subsequently recruits the Rap1-GTP–interacting adaptor molecule (RIAM) and talin, which binds to the β subunit of the integrin, causing separation of the subunits and extension of the integrin, thereby promoting a high affinity for ligands. Simultaneously, kindlin, recruited with the influence of PIP2, contributes to stabilizing and maintaining the integrin in its open conformation (Hyduk and Cybulsky, 2009; Sun et al., 2022; Pang et al., 2023).

**FIGURE 1:**
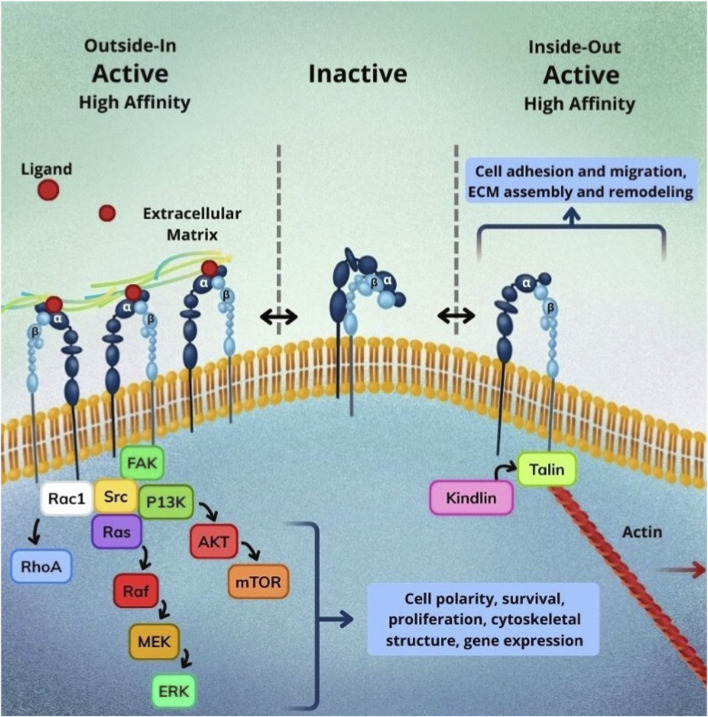
Overview of the bidirectional signaling of integrins and its impacts on extracellular and intracellular environments. Integrins exist in dynamic equilibrium between inactive and active conformations. In the inside-out pathway (right), intracellular adaptors such as talin and kindlin bind to the cytoplasmic tail of the β-subunit, inducing a conformational change that increases ligand affinity and promotes cell adhesion, migration, and ECM remodeling. Conversely, outside-in signaling (left) is triggered upon ligand binding to the extracellular α/β heterodimer, leading to activation of FAK/Src, PI3K/AKT/mTOR, and Ras/MEK/ERK cascades, as well as Rac1 and RhoA-mediated cytoskeletal remodeling. Together, these coordinated mechanisms regulate cell polarity, proliferation, survival, and gene expression in response to environmental cues.

In contrast, *outside-in* signaling occurs when the integrin binds to its ligand, for example, when integrin α4β1 interacts with VCAM-1. This binding activates focal adhesion kinase (FAK) and Src family kinases, which form a signaling complex that initiates multiple downstream cascades. The PI3K/AKT pathway contributes to cytoskeletal reorganization, facilitating cell migration (Xu et al., 2023); the MAPK/ERK pathway promotes cell survival by protecting leukocytes from apoptosis (Sun et al., 2015); and the Rho GTPase pathway regulates the formation of lamellipodia and filopodia, both of which support cell migration (Ridley, 2015). A summarized overview is presented in Figure 1.

Among the integrin family, the *ITGA4* gene has attracted particular interest because it encodes the α4 integrin subunit. This subunit pairs with β1 to form α4β1 [very late antigen-4 (VLA-4)] and with β7 to form α4β7, both of which are critical for lymphocyte adhesion, migration, and immune cell trafficking across the blood–brain barrier (BBB) and inflamed tissues (Yednock et al., 1992; Johnston and Kubes, 1999; Chigaev and Sklar, 2012; Pietronigro et al., 2019). Dysregulation of these interactions is strongly implicated in autoimmune conditions such as multiple sclerosis (MS), inflammatory bowel disease (IBD), and rheumatoid arthritis (O’Doherty et al., 2007; Glatigny et al., 2011; De Lange et al., 2017; Feng et al., 2025). Furthermore, the α4 integrin subunit is the pharmacological target of natalizumab. By binding to α4 integrins, natalizumab blocks the interaction of VLA-4 with vascular cell adhesion molecule-1 (VCAM-1) on the vascular endothelium, thereby preventing lymphocyte adhesion and transmigration across the BBB. This mechanism underlies its efficacy in MS and Crohn’s disease (Polman et al., 2006; Targan et al., 2007). However, interindividual variability in therapeutic response indicates that genetic and molecular factors regulating *ITGA4* expression and signaling may influence treatment efficacy and safety. Importantly, in diseases such as MS, variability in therapeutic response also reflects differences in disease course and broader clinical, genetic, and immunopathological heterogeneity (Lassmann et al., 2001; Engelhardt et al., 2022; Khan et al., 2025).

The data and information presented in this article were obtained through a comprehensive literature search primarily conducted in the PubMed database, using keywords including *ITGA4*, *ITGA4* pharmacogenetics, *ITGA4* integrin signaling, *ITGA4 SNP*, and *ITGA4* variant. Only articles published in English were considered. This review aims to contextualize ITGA4 biology, its historical perspective, and emerging pharmacogenetic insights in immune-mediated diseases.

## 2 *ITGA4* historical perspective

The discovery of *ITGA4* in the early 1990s marked a milestone in immunology and neurobiology. Initially identified as part of the VLA family on lymphocytes (Takada et al., 1989; Rosen et al., 1991). In humans, the gene was mapped to the q31-32 region of chromosome 2, while in rats, it was located on chromosome 3, indicating it is not syntenic between the two species. It is worth noting that *ITGA4* was the first example of a gene mapped to these two specific chromosomes (Zhang et al., 1991; Szpirer et al., 1992). Still in the 1990s, studies showed that α4 integrins were essential for T cell entry into the brain parenchyma and gut mucosa (Baron et al., 1993; Meenan et al., 1997). Landmark preclinical studies demonstrated that administration of antibodies targeting α4β1 integrin effectively prevented the development of experimental autoimmune encephalomyelitis (EAE), a widely used murine model of MS (Yednock et al., 1992). These findings established α4β1 as a critical mediator of lymphocyte trafficking into the central nervous system (CNS) and laid the foundation for integrin-targeted therapies in MS.

These discoveries positioned *ITGA4* within the broader context of the integrin family and helped to establish the conceptual framework for understanding integrin-mediated immune regulation. Since then, several integrin inhibitors have reached the clinic, including the small molecule αIIbβ3 antagonists tirofiban (approved in 1999) and eptifibatide, as well as multiple monoclonal antibodies targeting leukocyte integrins (ESPRIT Investigators. Enhanced Suppression of the Platelet IIb/IIIa Receptor with Integrilin Therapy, 2000; Gibson et al., 2002; Ley et al., 2016).

Among the integrin targets investigated, *ITGA4* emerged as one of the most promising because of its critical role in regulating immune cell trafficking. This insight paved the way for the development of natalizumab, a humanized monoclonal antibody against the α4 subunit, approved in the early 2000s as the first integrin inhibitor to achieve major therapeutic success in both neurology and gastroenterology, notably in relapsing–remitting MS and Crohn’s disease (Miller et al., 2003; Sandborn et al., 2005; Polman et al., 2006). Despite its clinical success, safety concerns, most notably the risk of progressive multifocal leukoencephalopathy (PML), highlight the challenges inherent to modulating immune cell trafficking (Kleinschmidt-DeMasters and Tyler, 2005; Van Assche et al., 2005; Bloomgren et al., 2012). In the mid-2010s, additional α4 integrin-targeting antibodies have been developed, particularly for IBD. Among these, vedolizumab (anti-α4β7) represents a newer generation of therapy designed to refine therapeutic selectivity and minimize adverse events such as PML, illustrating the ongoing evolution of integrin-targeted strategies beyond natalizumab (Feagan et al., 2013; Colombel et al., 2016; Arijs et al., 2018). As of 2023, ClinicalTrials.gov, ClinicalTrials.eu, AustralianClinicalTrials.gov.au and ChineseClinicalTrialRegistry.org.cn listed around 90 clinical trials involving integrin-targeting therapies, including integrin antagonists and imaging agents (Pang et al., 2023). Within this broader context, the integrin α4 subunit emerged as a particularly relevant target in immunology and neurology.

While therapeutic advances have transformed the clinical landscape, parallel efforts sought to elucidate the genetic architecture of *ITGA4* and its regulatory mechanisms. Genetic studies also began to emerge in the mid-1990s, the first sequence variants of the gene were published in 1995. Among the identified changes was a non-synonymous variant resulting in a nucleotide substitution from G to A in the coding region. This substitution was found to be associated with the deletion of the *Msp I* restriction site, converting the codon for arginine into glutamine. However, no functional alteration resulting from this variant could be identified (Szabo and McIntyre, 1995).

Recent genomic and functional studies have further refined our understanding of *ITGA4*. Genome-wide association studies (GWAS) and deep sequencing have identified regulatory variants that influence α4 integrin expression and splicing, some correlating with susceptibility to autoimmune diseases and response to natalizumab (De Lange et al., 2017; Demela et al., 2023; Clarelli et al., 2024). In parallel, multi-omic analyses of lymphocytes from patients with MS and IBD have revealed context-dependent modulation of *ITGA4*, highlighting its dynamic role in immune cell trafficking and providing opportunities for precision-targeted integrin therapies (Demela et al., 2023; Mennillo et al., 2024).

## 3 Clinical relevance of *ITGA4* genetic variants

Several studies have investigated potential associations between Single Nucleotide Polymorphisms (SNPs) in the *ITGA4* gene and the pathogenesis of various diseases, aiming to validate potential predictors of increased susceptibility or resistance factors. Many of these studies are conducted in MS patients that are the most prevalent autoimmune disorder that affects the CNS (Thompson et al., 2018). The first study investigating the impact of genetic variations in *ITGA4* on the risk of MS was conducted in a cohort of 352 patients from the Basque Country and 1,119 patients from Nordic countries. The aim was to compare the genotypic frequencies between patients and controls using both pyrosequencing and MALDI-TOF mass spectrometry. The following SNPs were analyzed: rs155141, rs1449263, rs3770138, rs3770136, rs3770132, rs155106, rs1038034, rs2305586, rs3770115, rs3816521, rs3770111, rs6714061, and rs4667319. Although none of the variants exhibited a significant association before correction for multiple comparisons, the genotype distribution of SNP rs1449263 showed a suggestive signal in the Basque cohort (O’Doherty et al., 2007).

In the following years, several groups examined alternative polymorphisms and expanded genetic analyses to other European populations. Nevertheless, population-specific effects and limited replication highlight a major challenge in consolidating these associations. For instance, the rs1143676 and rs113276800 polymorphisms showed no association in Italian patients (Andreoli et al., 2007), whereas in a Slovak cohort rs1143676 appeared more frequent in MS cases, but rs113276800 remained non-significant (Ďurmanová et al., 2015). More recently, the rs6721763 variant was associated with MS severity due to a prevalence in patients with a higher Multiple Sclerosis Severity Score (MSSS) in 389 greek patients (Dardiotis et al., 2017), and additional analyses in the same population identified seven SNPs (including rs12988934, rs11694175, and rs6721763) significantly associated with MS susceptibility (Dardiotis et al., 2019). Overall, these findings suggest that *ITGA4* genetic variation may influence both disease susceptibility and clinical severity in MS.

The connection between *ITGA4* polymorphisms and IBD has also been explored. In a landmark GWAS, involving nearly 60,000 individuals, researchers identified multiple loci associated with IBD, including SNPs within *ITGA4*, *ITGB8*, *ITGAL*, and *ICAM1*. Specifically, the authors found that variants in *ITGA4* were significantly correlated with changes in gene expression following immune stimulation, suggesting functional consequences in inflammatory pathways. This finding is particularly important considering the mechanism of action of vedolizumab and etrolizumab, which target the α4β7 integrin heterodimer (encoded by *ITGA4* and *ITGB7*) to modulate gut-selective immune responses in IBD treatment (De Lange et al., 2017).

Associations with other neurodevelopmental and neurodegenerative conditions have also been investigated. The rs155100 SNP was significantly associated with autism, showing a higher prevalence in a cohort of 164 Portuguese patients compared to controls (Correia et al., 2009). Additionally, another study identified a significant association between the rs12690517 SNP and the prevalence of autism in a cohort of 179 Irish patients (Conroy et al., 2009). To date, only one study has evaluated the association between *ITGA4* variants and susceptibility to Alzheimer’s disease. This study demonstrated a significantly higher prevalence of the AG genotype of rs1143676 in a cohort of 104 Slovak patients compared to controls (Durmanova et al., 2018).

## 4 Pharmacogenetics of *ITGA4*

There is a growing list of genetic polymorphisms in drug targets that have been shown to influence drug response (Eichelbaum et al., 2006). Although no drug-gene interactions involving *ITGA4* are currently recorded in the PharmGKB database, the gene has received increasing attention due to its therapeutic relevance in immune-mediated diseases. Recent studies have begun to explore the influence of *ITGA4* polymorphisms on drug response, particularly in the context of biologic therapies used to treat immune-mediated conditions such as MS and IBD. Despite these efforts, the number of pharmacogenetic investigations directly addressing *ITGA4* remains limited, highlighting a substantial knowledge gap that complicates the development of personalized therapeutic strategies for patients treated with *ITGA4*-targeted agents.

A study conducted in a cohort of 50 Italian MS patients, including 22 responders and 28 non-responders to interferon-beta (IFN-beta) therapy, aimed to assess *ITGA4* expression in both groups. The results revealed a reduction in *ITGA4* expression in responder patients, while non-responders exhibited unchanged expression levels (Muraro et al., 2004). A study conducted in a cohort of 70 Iraqi MS patients demonstrated a higher prevalence of the AG genotype of rs1143676 SNP in patients who did not respond to natalizumab treatment. This suggests that the AG genotype could potentially serve as a biomarker for therapeutic failure (Ahmed et al., 2023a). Another similar study conducted by the same group using the same cohort aimed to assess the association between the rs200000911 variant and the therapeutic outcome of natalizumab. No significant associations were found, including two additional intronic variants identified through Sanger sequencing: rs936587744 and rs2305588 (Ahmed et al., 2023b).

An interesting study using *in silico* approaches explored the pharmacogenetic impact of the rs200000911 SNP, a lysine-to-arginine (K256R) transversion at amino acid position 256 in the *ITGA4* protein (Cavaliere et al., 2017). Given its location within the ligand-binding region, this variant was hypothesized to affect drug binding, especially for natalizumab. However, the results showed that rs200000911 may not contribute to failure mechanisms in non-responding patients. Nevertheless, this study exemplifies the utility of *in silico* tools in pharmacogenetics as a rapid, cost-effective screening method for prioritizing SNPs for experimental validation and clinical investigation.

Although still limited, pharmacogenetic studies have begun to identify specific *ITGA4* variants that may influence risk of adverse effects. A notable example is the rs3770126 SNP, identified through a GWAS investigating renal toxicity in HIV-positive Southern African individuals undergoing treatment with tenofovir. While the study primarily focused on polymorphisms in transporter genes such as *ABCC4* and *ABCC10*, *ITGA4* rs3770126 emerged as one of the top genome-wide signals (P = 6.1 × 10^−7^), pointing to a potential association with drug-induced renal dysfunction. Although this association did not withstand correction for multiple comparisons, the finding underscores the possibility that *ITGA4* may influence pharmacological pathways beyond its well-established role in immune cell trafficking (Mateza et al., 2023). Table 1 summarizes all clinical and pharmacogenetic associations found regarding *ITGA4* SNPs.

**TABLE 1:** Summary of clinical and pharmacogenetic associations related to *ITGA4* SNPs.

SNP	Association	References	Type of study	*p-value*
Clinical relevance				
rs155141, rs1449263, rs3770138, rs3770136, rs3770132, rs155106, rs1038034, rs2305586, rs3816521, rs3770111, rs6714061, rs4667319	MS susceptibility	O’Doherty et al. (2007)	Case-control	0.136, 0.098, 0.973, 0.818, 0.136, 0.542, 0.942, 0.748, 0.212, 0.857, 0.929, 0.505, respectively
rs1143676, rs113276800	MS susceptibility	Andreoli et al. (2007)	Case-control	0.994, 0.454, respectively
rs1143676, rs113276800	MS susceptibility	Ďurmanová et al. (2015)	Case-control	0.04 for the GG genotype, 0.36, respectively
rs6721763	MS severity	Dardiotis et al. (2017)	Case-control	3.00 × 10^−06^
rs12988934, rs11694175, rs17224277, rs155103, rs17225354, rs11689738, rs6721763	MS susceptibility	Dardiotis et al. (2019)	Case-control	0.016, 0.005, 0.0009, 0.007, 0.0036, 0.0051, 9.999e-005, respectively
rs155100	Autism prevalence	Correia et al. (2009)	Case-control	0.019
rs12690517	Autism prevalence	Conroy et al. (2009)	Case-control	0.008
rs1143676, rs113276800	Alzheimer susceptibility	Durmanova et al. (2018)	Case-control	0.04 for dominant and codominant models, 1.00, respectively
rs6740847	IBD susceptibility	De Lange et al. (2017)	GWAS	1.22 × 10^−13^
Pharmacogenetic relevance				
rs1143676	Therapeutic failure to natalizumab	Ahmed et al. (2023a)	Case-control	0.024
rs200000911, rs936587744, rs2305588	Therapeutic failure to natalizumab	Ahmed et al. (2023b)	Case-control	0.896, 0.975, 0.063, respectively
rs200000911	Therapeutic failure to natalizumab	Cavaliere et al. (2017)	*In silico*	-
rs3770126	Adverse effects to tenofovir treatment	Mateza et al. (2023)	Cohort	6.1 × 10–^7^

Taken together, available evidence indicates that *ITGA4* genetic variants and expression patterns may have relevance in autoimmune and neurodevelopmental contexts. While findings in MS are the most robust, results remain inconsistent across cohorts. Broader studies with larger, well-characterized populations and integrative approaches combining genetics, transcriptomics, and functional assays will be essential to clarify the role of *ITGA4* as a biomarker or therapeutic target.

## 5 Discussion

Since the first descriptions of the α4 integrin and the *ITGA4* gene in the 1990s, there has been a substantial effort to identify genetic variants that could serve as feasible clinical and pharmacogenetic biomarkers capable of predicting clinical outcomes such as disease susceptibility and severity, as well as therapeutic outcomes such as drug efficacy and adverse effects. Over the years, studies have been conducted across various disease contexts, including Alzheimer’s disease, autism, and IBD; however, the primary focus has remained on MS, the most prevalent autoimmune disease affecting the CNS due to the critical role of integrin α4β1 in its pathogenesis, as it is fundamental for leukocyte migration across the BBB (Hočevar et al., 2019; Ahmed et al., 2023a).

Although several SNPs have been associated with clinical outcomes in these conditions, inconsistencies among findings persist, underscoring the need for replication and validation in larger and more ethnically diverse patient cohorts. Despite the growing interest in *ITGA4* pharmacogenetics, several challenges hinder its translation into clinical practice. Key issues include inconsistent evidence regarding the functional relevance of specific polymorphisms and the scarcity of functional studies elucidating how *ITGA4* variants mechanistically influence gene expression, protein function, cellular behavior, or drug binding. Understanding these molecular mechanisms is essential for interpreting the clinical significance of genetic variation and for identifying variants that may serve as reliable biomarkers of treatment response or risk of adverse effects (Grossman and Miller, 2010; Hočevar et al., 2019).

Beyond leukocyte adhesion, VLA-4 signaling activates several downstream cascades, including PI3K/AKT, MAPK, and RhoA/ROCK pathways, which regulate cytoskeletal reorganization, cell survival, and migration (Hehlgans et al., 2007; Schmid et al., 2013). Variants within *ITGA4* gene could potentially alter integrin affinity or intracellular signaling capacity, thereby modulating these downstream events. However, associations involving *ITGA4* tend to display greater population-specific variability, possibly reflecting differences in ethnic genetic background, sample size, environmental modifiers (e.g., vitamin D status or viral exposures), and genotyping methodologies (O’Doherty et al., 2007; Oksenberg and Baranzini, 2010; Beecham et al., 2020). Such discrepancies emphasize the need for multiethnic cohorts and standardized genotyping and expression of quantification strategies to achieve reproducible findings.

Furthermore, the integration of *ITGA4* research into personalized medicine remains limited. Given its pivotal role in immune cell trafficking and chronic inflammatory diseases, *ITGA4* represents a promising target for precision therapies. Future studies should adopt multidisciplinary approaches combining *in silico* modeling, transcriptomics, and clinical data to validate variants, elucidate underlying mechanisms, and identify actionable biomarkers that can guide individualized treatment strategies.
